# Patient Preparation in Walk-in Clinics Through the Implementation of a Consultation Preparation Sheet: Qualitative Study

**DOI:** 10.2196/83489

**Published:** 2026-10-08

**Authors:** Juliette Borges, Claude Richard, Marie-Hélène Goulet, Marie-Eve Lavoie, Viviane Tran, Clara Dallaire, Marie-Thérèse Lussier

**Affiliations:** 1 Communications Department, Université de Montréal, C.P. 6128, succursale Centre-ville, Montreal, QC, H3C 3J7, Canada, 1 514 662 3138; 2 CHUM: Centre de recherche du Centre hospitalier universitaire de Montréal, Montreal, QC, Canada; 3 Faculté des sciences infirmières, Université de Montréal, Montreal, QC, Canada; 4 Centre de recherche de l’Institut universitaire en santé mentale de Montréal, Montreal, QC, Canada; 5 Faculté de Médecine, Université de Montréal, Montreal, QC, Canada; 6 Centre de recherche Azrieli du CHU Sainte-Justine, Montreal, QC, Canada

**Keywords:** patient participation, physician-patient relations, primary health care, patient engagement, communication tools, qualitative research, implementation science, patient-centered care

## Abstract

**Background:**

Effective communication is essential for high-quality care, yet in walk-in clinics, patients often have limited time to prepare, and physicians face challenges in understanding patients’ needs due to lack of prior contact. To address this, a consultation preparation sheet (CPS) was developed to help patients articulate their symptoms, concerns, and expectations.

**Objective:**

This study aimed to explore the implementation and perceived usefulness of a CPS designed to support patient preparation during walk-in clinic consultations.

**Methods:**

We conducted a qualitative exploratory study using semistructured interviews and focus groups, with data collection informed by the RE-AIM (Reach, Effectiveness, Adoption, Implementation, and Maintenance) framework and the PACE (prepare, ask, check, and express) method. The CPS was implemented over 9 months (January-October 2019) in a university-affiliated walk-in clinic in Quebec, Canada. During this period, 3115 patients received the CPS in the waiting room. Of the 23 patients who expressed interest in further participation, 12 were recruited for interviews. Additional participants included 2 physicians, 2 administrative staff, 2 nursing assistants, and 1 clinic manager. In total, 6 interviews and 5 focus groups were conducted. Data were transcribed verbatim and thematically analyzed, with AI-assisted review used as a supplementary check on data coverage.

**Results:**

Patients generally found the CPS helpful for organizing their thoughts, reducing stress, and avoiding omissions during consultations. It was considered particularly useful for patients with multiple concerns or communication difficulties. However, physicians rarely referred to the CPS, often preferring direct verbal exchanges. This lack of acknowledgment sometimes frustrated patients and diminished their perception of its usefulness. Organizational challenges—including inconsistent distribution, lack of explanation, and an unclear workflow—further hindered implementation. Participants suggested clearer communication about the CPS’s original purpose, earlier access through electronic formats, and brief clinician acknowledgment when patients chose to share their priorities.

**Conclusions:**

The CPS shows promise as an optional patient-preparation tool for walk-in consultations. Earlier access and a clear explanation may support its use without requiring formal clinician review; brief acknowledgment may suffice when patients choose to share their priorities. Future studies should assess improved practice implementation strategies and digital adaptations.

## Introduction

### Background

Physician-patient communication is central to disease management, relying on mutual collaboration, in which physicians provide medical expertise and patients share their experience of the illness [1,2]. Effective collaboration requires dialogue to identify health issues and their impact on the patient’s life [3]. Beyond biomedical aspects, patient-centered communication enhances information retention, treatment adherence, stress management, and trust in the health care system [4-10].

Effective clinical communication depends in part on patients’ ability to contribute actively to the consultation, particularly by providing information to physicians for accurate diagnosis and treatment [11]. Cegala [12] defines participation through 4 dimensions: seeking information, stating preferences or opinions, providing information, and expressing concerns. This approach helps physicians address patients’ ideas, fears, and expectations. However, barriers such as wait times, consultation duration, health literacy, language, and trust levels affect participation [2,12-15].

Studies indicate that methods and tools supporting patient preparation improve question-asking and information retention without extending consultations [2,7,12,16-18]. These strategies take various forms. For instance, Cegala et al [19] developed the PACE (prepare, ask, check, and express) method to help patients organize their concerns during consultations. Other tools, like DocVisitGuide and question prompt lists, help guide the patient before, during, and after consultations [20,21]. However, these resources require prior training, which can be both costly and time-consuming, whereas online tools tend to offer more accessible alternatives [22-25]. In this context, *Discutons Santé*, a PACE-based website, was developed to enhance chronic disease management [22,25,26]. Users have reported increased engagement, reduced stress, and improved consultation readiness [25].

In addition to previously reported barriers to the use of web-based tools, such as limited digital literacy and internet access for some users [27], walk-in consultations pose challenges for *Discutons Santé* adoption. In Quebec and Canada, walk-in clinics provide quick access to primary care, especially for those without family physicians [28-31], reducing emergency department pressure [32]. However, this type of consultation may limit patient preparation time and weaken physician-patient relationships. The physician’s lack of knowledge of the patient’s history, as well as the absence of an established and ongoing trusting relationship between physician and patient, can weaken the quality of communication about the illness and needs [28,30,31].

To address these limitations, the consultation preparation sheet (CPS) was introduced in 2018, inspired by the *Discutons Santé* website and adapted for walk-in clinic patients. While there is no research, to our knowledge, on optimizing walk-in consultation exchanges, this study examines CPS implementation in a Quebec walk-in clinic and its perceived impact on consultation quality.

### Study Objectives

Our objectives were to explore (1) participants’ perceptions of barriers and facilitators to the implementation of CPS, (2) participants’ perceptions of the characteristics, impact, and usefulness of CPS when consulting in walk-in clinics, and (3) participants’ interests in the future use of the CPS, and suggestions for improving its implementation.

## Methods

### Study Design

This study is part of a broader mixed methods research design. The present paper reports the qualitative component, following COREQ (Consolidated Criteria for Reporting Qualitative Research) principles [33], with the completed COREQ checklist provided in Checklist 1.

### Study Setting

The CPS was implemented over 9 months (January 6-October 4, 2019) in a walk-in clinic of an academic family medicine clinic affiliated with the Université de Montréal. In this clinic, family medicine residents see many patients and are supervised by attending physicians. This clinic, serving approximately 15,000 registered patients as of March 2019, was already familiar with and promoted the use of the *Discutons Santé* website [25].

### Codevelopment and Pretesting of the CPS

Available in French and English (Multimedia Appendix 1), the CPS was codeveloped with patients and physicians and then pretested with 25 patients consulting at the hospital where the study is set (13 from the walk-in clinic and 12 from the emergency room of the hospital near the walk-in clinic) and 15 physicians from the same hospital (7 working in the walk-in clinic and 8 in the emergency room) to assess and refine its relevance, format, and content. Administrative staff contributed to its implementation evaluation but not to its initial design.

The CPS comprises 10 multiple-choice and 6 open-ended questions. It was designed for approximately a 6th grade reading level to facilitate completion in the waiting room, including patients with more limited literacy skills.

The CPS covers both the biomedical and experiential dimensions of semiurgent consultations: (1) reason for consultation, (2) patient concerns, (3) expectations from the consultation, and (4) discomfort level and impact on daily activities.

### Implementation Process

This pragmatic implementation project was first defined collaboratively by the project coordinator, the clinic manager, and a nursing assistant. No formal training was developed by the project team for clinicians (physicians, family medicine residents, and nurses); however, they were informed that the CPS would be offered to patients. The responsibility for introducing the tool to all clinicians was left to the clinic manager, who had flexibility in how this was carried out. In practice, the tool was typically introduced during regular weekly team meetings. Upon arrival at the clinic and confirming their presence with the administrative staff at reception, patients were invited to complete the CPS in the waiting room before seeing the physician. Administrative staff were responsible for distributing the CPS (an example script for explaining the CPS to patients was provided). Patients were encouraged to share the CPS with their physician if they wished, while physicians were encouraged (but not mandated) to review its content when presented by patients.

### Participants and Recruitment Process

#### Sample Size and Recruitment

Given the exploratory nature of this qualitative study, no a priori sample size was given in advance. Recruitment followed a pragmatic approach: all eligible participants who expressed interest and could be scheduled within the study time frame were invited to take part. The final sample was therefore shaped by participant willingness, availability, and the time constraints of the implementation project, rather than by a predefined numerical target. The aim was not statistical representation, but to obtain sufficiently rich accounts from the different participant groups involved in the implementation of the CPS.

#### Patients

Of the 4297 patients invited to participate, 3115 (72.5%) received the CPS in the waiting room prior to their consultation. Between August and October 2019, an optional appreciation questionnaire was also offered. Its purpose was to gather patients’ impressions of the CPS and to allow them to indicate their interest in joining a focus group or participating in a semistructured interview.

Eligibility criteria included having previously completed the CPS, being 18 years of age or older, and being able to read and speak either French or English. Two hundred patients received the appreciation questionnaire. Of the 23 who expressed interest in participating further, 12 were ultimately recruited for interviews, including 8 men and 4 women. The reduced number was due to some patients later declining, being unreachable, or too much time having passed since their initial expression of interest.

#### Health Care Professionals and Administrative Staff

Physicians, nurses, administrative staff, and a manager were invited to take part in a focus group or a semistructured interview at the end of the project to share their experiences, with eligibility requiring that participants work at the clinic during the CPS implementation and attend to patients in walk-in consultations during the project. Physicians and nurses were approached through the administrative manager and via email by the research team, while administrative staff were approached through the administrative manager. The clinic manager was approached in person. The research team was not provided with information on the number of eligible staff who did not participate or the reasons for nonparticipation; no information on participant withdrawals was available. Participants included 2 family physicians (a woman with 11 y and a man with 3 y of clinical experience), 2 female nurses employed at the clinic for over 10 years, 2 female administrative staff members with more than 15 years of experience at the clinic, and a male clinic manager aged between 40 and 50 years who had occupied this position for 5 years.

### Ethical Considerations

The study was approved by the Scientific and Ethics Committee of the Laval Health and Social Services Center (study: 2018‐2019/01-03-E).

Before interviews and focus groups, all participants provided written consent, and every effort was made to protect participants’ confidentiality. Only the interview data are presented here.

Participants received a snack during focus groups but no financial compensation.

### Data Collection

#### Interviews and Focus Groups

Interviews and focus groups were conducted by MEL, female, who was a research associate at the time of the study and held a PhD. She had more than 15 years of experience and training in qualitative interviewing. No relationship had been established with participants before recruitment. Participants were informed of the interviewer’s role and the purpose of the study and were reminded before each interview that the discussion would focus on their experience with the CPS.

Between October 2019 and January 2020, interviews and focus groups were scheduled based on participant availability. We held sessions with health care professionals (nurses and physicians), the manager, and administrative staff in a private clinic room, ensuring confidentiality. We conducted patient focus groups in the same setting, while those unable to attend participated in individual telephone interviews. No one other than the participants and the researcher conducting the interview or focus group was present during the sessions.

In total, we conducted 6 semistructured interviews (1 in-person interview with the administrative manager and 5 telephone interviews with patients) and 5 in-person focus groups. These included physicians (focus group 1), administrative and nursing staff (focus group 2), and patients (focus groups 3 to 5). These were distributed as described in Figure 1. Sessions lasted approximately 30 to 60 minutes, for a total of 6 hours of audio-recorded material. All sessions were recorded and transcribed verbatim; no formal field notes were taken during or after the interviews or focus groups. No repeat interviews or follow-up interviews were conducted. Transcripts were not returned to participants for comment or correction, and participants were not asked to review or validate the study findings.

**Figure 1.**
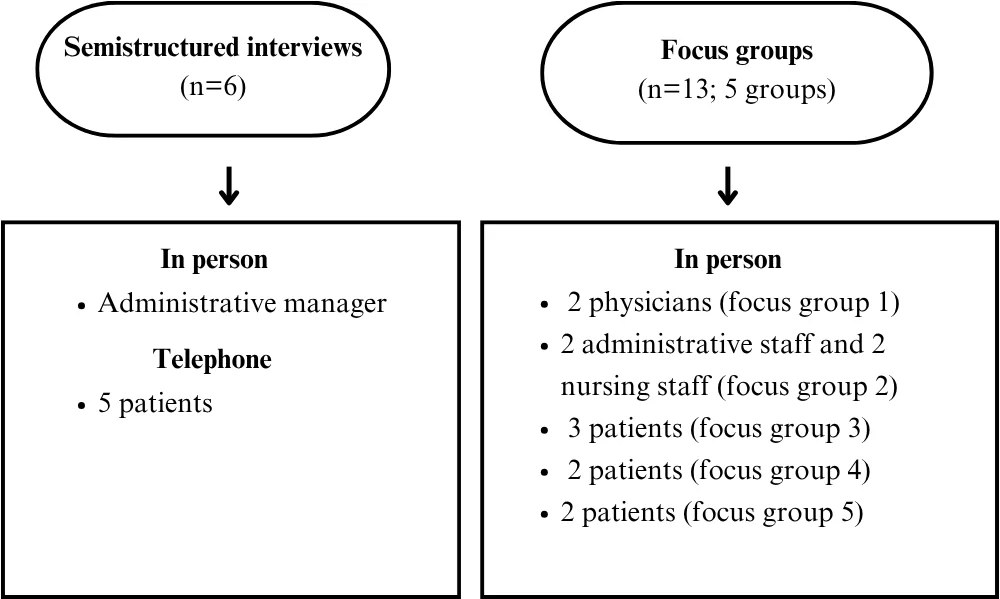
Participant distribution by role and method.

#### Interview Guides

Patient and physician interview guides (Multimedia Appendix 2) largely followed the PACE approach [19]. Patient discussions explored prior knowledge of the *Discutons Santé* website, first impressions of the CPS, its use in consultation (consideration of the CPS by the physician, verification of information, description of symptoms, expression of concerns and questions to ask the physician, possible effects on stress reduction, an active role in the consultation, and structure of the encounter), interest in the future use of the CPS, and improvement suggestions. Physician interviews examined CPS walk-in clinic implementation, perceived usefulness, consultation and workflow impact, communication quality, interest in the future use of the CPS, and improvement suggestions.

For the manager, administrative, and nursing staff, interview guides largely followed the RE-AIM (Reach, Effectiveness, Adoption, Implementation, and Maintenance) framework, as described by Glasgow et al [34], which evaluates the impact of health interventions and their implementation into care. The discussions covered implementation challenges, perceptions of the CPS, workload impact, and improvement suggestions.

These frameworks served as sensitizing frameworks to guide data collection rather than to structure the analytic process or the presentation of results.

### Analysis

#### Thematic Analysis

To analyze participants’ experiences and perceptions regarding the CPS, a thematic analysis was conducted, following the steps described by Braun and Clarke [35,36] and listed below. This method allowed themes to be developed iteratively from both the study questions and the interview data. The following steps were followed: (1) the main analyst repeatedly listened to interviews and transcribed them during data familiarization. (2) The main analyst generated codes by matching verbatim responses to interview questions (predefined codes) and supplementing them with new codes coming from emerging topics spontaneously introduced by participants. (3) The main analyst searched for themes by grouping responses according to the type of participant (patients, clinicians, staff, or managers). (4) The main analyst reviewed and iteratively refined the themes, and the supervisor validated them. (5) Defining themes involved both the main analyst and the supervisor agreeing on the final themes.

The analysis did not rely on a formal coding tree developed in advance. Instead, it followed an iterative process of thematic construction based on the interview material. After each interview or focus group was transcribed and reviewed, its content was examined in relation to the study questions and to the themes already identified in the preceding material. When a participant introduced elements that had not yet been represented in the analysis, these were added to the evolving thematic structure. When material corresponded to themes already identified, it was used to enrich, nuance, or contrast those themes. In this way, the analytic process consisted of progressively building thematic presentations that brought together convergent, divergent, and complementary perspectives across interviews and participant groups. This process continued throughout the full corpus.

Thematic sufficiency was considered to have been reached when successive interviews no longer contributed substantively new thematic elements but rather added variation, illustration, or nuance to existing themes. One researcher, the main analyst (JB), performed the primary coding of the full dataset. No dedicated qualitative data-analysis software was used.

#### Supplementary AI-Assisted Data Coverage Check

In addition to regular discussions between the main analyst and the supervisor, ChatGPT-4o (OpenAI) was used as a limited supplementary tool for a language-based data coverage check during the analytic process, in line with recent studies exploring the potential contributions of this tool in qualitative and thematic analysis [37-39]. Specifically, it generated a separate categorization of excerpts to help identify potential blind spots, overlaps, omissions, or alternative ways of grouping the data. The AI output was not treated as an independent coder, and no interrater agreement was calculated or used as a marker of rigor. Instead, the research team compared the AI-generated output with the developing thematic structure and critically assessed whether any suggested distinctions or groupings were conceptually relevant and supported by the data. All interpretive decisions remained the responsibility of the research team. The AI-assisted review, therefore, served as a supplementary analytic sense-check intended to strengthen data coverage, not as a reflexivity or validation procedure. Importantly, ChatGPT was not used to generate theoretical interpretations.

We adjusted ChatGPT parameters to ensure participant confidentiality and made sure that no personally identifiable information was entered into the platform. We conducted the verification according to the following process: (1) Verbatim compilation involved compiling all responses, with the type of participant (patient, physician, manager, administrative staff, or nursing staff) identified for each statement. (2) The AI-assisted data coverage check involved ChatGPT generating an independent language-based categorization of the anonymized excerpts. (3) The comparison involved the research team comparing this output with the developing thematic structure. (4) The discrepancy review found high convergence for explicit content. Differences from implicit inferences were examined, discussed, and resolved by consensus between the main analyst and the supervisor. The necessary adjustments were made.

#### Research Team Positionality

This study was conducted by a multidisciplinary research team with expertise in health communication, primary care, and qualitative research. Some members of the team had previously been involved in the development and implementation of the *Discutons Santé* platform, from which the CPS was derived. This prior involvement provided in-depth knowledge of the intervention and its intended use, but it may also have shaped expectations regarding its relevance, feasibility, and uptake in clinical practice. To address this, particular attention was paid during data collection and analysis to participants’ critical, ambivalent, or negative perspectives on the CPS in order to avoid privileging confirmatory interpretations.

In addition, 3 members of the research team, including the main analyst (JB) and the patient partner (CD), had not been involved in the implementation of the project, which contributed to a more external and critical perspective on the data, while the involvement of a patient partner also ensured the integration of the patient voice in the interpretation of the findings. Interpretive decisions were discussed iteratively between the main analyst and the supervisor, and emerging themes were reviewed by all authors in light of both supportive and disconfirming cases.

## Results

### Summary

This research highlights key points regarding CPS implementation and use. Table 1 summarizes findings from the interviews. The themes are presented in greater detail in the section below, with illustrative quotes from participants.

**Table 1.** Summary of key themes across participants.

Themes	Patients	Physicians	Manager and administrative/nursing staff
Implementation of CPS^a^	Lack of explanation; often not shared with physicians	Low familiarity with the tool; lack of explanation	Increased administrative workload; unclear transmission process; unoptimized flow
Use in consultation	Rarely used	Rarely used; preference for verbal communication	Not systematically provided to physicians
Perception of benefits	Helps structure ideas; reduces stress	Useful for disorganized patients or with multiple issues; supports prioritization	Clarifies expectations
Barriers encountered	Ignored by physicians	Lack of time; limited implementation	Internal communication and organizational issues; low physician interest
Suggestions for improvement	Early access to CPS; electronic version	Early access to CPS; need for training and systematic implementation	Improve internal communication; need for more structured support

a CPS: consultation preparation sheet.

### Implementation of CPS in the Clinic

#### Workload

Administrative staff experienced an increased workload with CPS, especially when managing multiple forms. However, health care professionals (nurses and physicians) reported no significant impact on their workflow, stating that CPS implementation neither disrupted their activities nor changed their work approach.

#### Distribution and Circulation of the CPS

Interviews revealed internal organizational issues, particularly between health care professionals and administrative staff regarding managing the distribution and circulation of the CPS. Patients said the way they submitted their CPSs varied: 4 gave them to a nurse, 2 to a physician, and physicians interviewed sometimes found them on their desk. The limited time in the waiting room has been identified as a barrier by the manager, administrative, and nursing staff, making it difficult to distribute and fully complete the CPS before patients are called into the exam room. Consequently, some patients filled it out partially during consultations with a nurse.

*Sometimes they [patients] are with us, and the nurse already calls the patient before we finish giving the form […] some completed it while waiting after seeing the nurse.*

[Administrative staff 2]

Lack of communication and explanation about the CPS caused confusion among patients and physicians. While the manager said some physicians were interested in the tool, others seemed unaware of its existence and did not integrate it into their clinical routine. The 2 interviewed physicians noted that family medicine residents often asked questions about the CPS, indicating limited familiarity with the implementation of the tool. According to administrative and nursing staff, some physicians’ lack of interest affected follow-up efforts regarding the reception of CPS by physicians.

*And I’ve even had residents go, “What am I supposed to do with this?”—you know, they’d come back with the form like, “What do I do with this?” And I’d be like, “Well, it’s to see, you know, why the patient’s here, whether we’re going to meet their expectations—it helps guide the purpose of the consultation.*

[Physician 2]

Regarding the involvement of physicians, the manager mentioned a significant barrier: the clinic, which is affiliated with a regional hospital, was, at the time of the CPS implementation, undergoing its periodic 5-year accreditation by Accreditation Canada, so CPS was not seen as a priority.

According to the administrative and nursing staff, some patients also complained about having to complete the CPS during return visits. This task was demotivating for them when patients did not understand the purpose of the tool.

*Me, I think that once the form is gone, it’s really…our role is to hand it in, yeah, to check it, and bring it…to pass it on to the resident physician, but at that point, it’s up to them. And I’ve said it to them often. I went to see [manager] and I said, look, we’re being told they won’t read it and that they don’t even look at it.*

[Nurse]

*That’s not very motivating.*

[Administrative staff 1]

Overall, the manager and staff stressed the need for better project oversight and internal communication. As an implementation facilitator, clinician acknowledgment of the CPS was consistently described as important to successful implementation, with suggestions to leverage existing committees and to conduct regular surveys to assess team understanding.

### Characteristics of the CPS (Format and Content)

Despite organizational and communication limitations affecting the implementation of CPS in the clinic, as stated in the previous section, the CPS format and content received overall positive feedback from the manager, administrative, and nursing staff. Among the 12 patients interviewed, 8 were unfamiliar with the *Discutons Santé* website before the study. When asked about their reactions to the CPS, 5 expressed a positive response, while 3 reported confusion or avoidance, which they attributed to a lack of explanation regarding the CPS purpose.

*I was very pleasantly surprised. I’ve been going to the family medicine clinic for several years, and I discovered the form at the end of the summer. So it was my first experience with it, and my first impression was really, really positive.*

[Patient 2]

Patients and physicians found the CPS simple and quick to complete. 4 patients deemed the symptom-related questions relevant, while opinions were mixed regarding questions about concerns (1 found them useful vs 3 unnecessary), discomfort (3 useful vs 2 unnecessary), and consultation expectations (4 useful vs 4 unnecessary). One argument against the questions about concerns and expectations was that it seemed self-evident: people naturally worry when seeking care, and they expect to understand their condition and receive solutions.

*But of course [in response to the question about expectations], you want to be relieved, you want to know what you have. It seems logical to me—it’s automatic. If you're going to consult, it’s because you want to understand what’s going on.*

[Patient 3]

However, 1 patient cited format challenges, including literacy barriers and language selection.

*Like I was saying earlier, there might be an issue for people who are illiterate — you might need to find another way if you want to implement that document.*

[Patient 6]

Physicians found the CPS questions relevant in the walk-in context, particularly those regarding repeated consultations for the same issue.

### Perceived Impact and Usefulness of CPS in Consultation

#### CPS Benefits

Participants—including patients, physicians, managers, and staff—acknowledged that the CPS could help patients better prepare for consultation. In particular, it would allow them to structure their thoughts and avoid forgetting important points to discuss. Five patients found it useful for organizing their concerns and communicating effectively.

*I would say that I was better equipped thanks to the CPS. When you're better equipped, you feel more at ease, you communicate more easily with your doctor, so in that sense, it was beneficial.*

[Patient 2]

Some patients reported positive effects, including stress management and the ability to better identify their needs and level of discomfort. However, 4 patients did not find the CPS beneficial, citing reasons such as the physician’s disregard for it, a consultation for a baby where the CPS was deemed inappropriate, or health issues considered too simple or obvious.

Beyond individual experiences, the majority of patients (n=8) recognized broader advantages: avoiding omissions (n=6) and taking a more active role in consultations (n=5). Two patients highlighted increased patient responsibility:

*There is certainly an awareness of the responsibility we have as patients. That is, we realize…it’s not true that the doctor can know everything. They could run a full set of tests while we remain completely silent, but they need to talk to us because our lived experience is essential to properly complete the consultation. So, I think it fosters a sense of responsibility in that regard […].*

[Patient 11]

Half of the patients interviewed (6/12) recognized that preparation is crucial for effective consultations, and 2 generally considered themselves to be well prepared. Three mentioned that they saw CPS as particularly useful for children, older adults, or those with communication or memory difficulties.

Physicians, unfamiliar with patients before consultations, found it difficult to assess the CPS’s impact but acknowledged its role in addressing concerns and managing expectations. They also noted that some patients relied on the CPS to make sure they didn’t forget anything. Finally, they acknowledged that the CPS could be useful for structuring discussions during consultations, particularly with patients who have multiple issues or a disorganized presentation in the context of walk-in clinics:

*[…] In consultations, as we were saying, walk-ins are often focused on a single issue. Sometimes, a patient comes in with what we call a ‘grocery list’—five, six, seven, or even eight problems. Being able to prioritize them a little, to see what is most urgent or not, already gives the patient a useful exercise […]. So, I think it’s beneficial in a walk-in setting […].*

[Physician 1]

According to the manager, while physicians were divided on the CPS’s value, those who used it noted time-saving benefits. Administrative and nursing staff emphasized its role in clarifying patient expectations and facilitating collaboration on health issues, especially during first-time visits.

#### Barriers to CPS Use During the Consultation

Despite its advantages, among the 12 patients interviewed, 8 did not show the CPS to their physician, citing reasons such as perceived physician disinterest (n=1), discomfort with the physician (n=1), or the belief that their issues were self-evident (n=2). Only 1 patient actively presented and discussed the CPS during the consultation. Although most patients did not bring it up themselves, many were disappointed that the CPS was not acknowledged. Eleven patients observed that their physicians did not refer to it at all or did so only rarely during consultations. This lack of consideration led to varied reactions: 5 patients expressed surprise, disappointment, or frustration, while 3 felt that completing the CPS had been redundant or unnecessary.

*The doctor has to get that to use this, they need to read it, they need to be interested in reading it, definitely. If this doesn’t get read, then there’s no point to the whole thing.*

[Patient 1]

Physicians confirmed infrequent use of the CPS and expressed preferring direct verbal interactions with the patient. All participants stressed that physician engagement was important to avoid patient frustration. Three patients found completing the CPS beneficial regardless of physician use, but 5 questioned its relevance if not acknowledged. Two stated that neglecting the CPS negatively impacted the physician-patient relationship.

*The most important thing is the physician, because it is the contact between the physician and the patient. I think both of them should get to know each other better. […] It wasn't my family doctor, I understand that, but I expected him to read […].*

[Patient 9]

### Future Use of the CPS and Suggestions for Improving Its Implementation in Clinical Routines

Most participants supported the future use of the CPS. Seven patients expressed their intention to continue using it, particularly when consulting unfamiliar physicians, addressing new health concerns, or during infrequent visits. In contrast, 3 participants found the CPS less relevant for routine appointments with their regular physicians.

Participants proposed several ways to enhance its implementation into clinical routines. A key suggestion shared among participants was the need for clearer communication—to ensure that all stakeholders understand the CPS’s purpose, benefits, and link to the *Discutons Santé* website. Patients also expressed a preference for accessing and completing the CPS before their clinic visit, allowing physicians to review it in advance and be better prepared for the consultation.

*In most cases, I would say I would like to have a sheet before leaving in the morning […] to make a summary of what I’m going to talk to them about, the issues I have, and everything.*

[Patient 5]

*So, having the CPS before going to the clinic?*

[Interviewer]

*Mmmhm. [And] for the doctor to have time to review it.*

[Patient 5]

Electronic access was widely suggested, including via mobile apps, appointment confirmation links, or interactive waiting-room kiosks. Physicians proposed centralized digital platforms or physical drop-off points near their desks to improve accessibility. They recommended providing the CPS directly to physicians rather than to family medicine residents, in order to encourage greater involvement from physicians.

### Enhancing Adoption and Promotion

#### Participants’ Suggestions for CPS Implementation

The participants’ suggestions can be grouped into 3 main categories: better explanation of the CPS, better adoption of CPS usage by clinicians and administrative staff, and promotional tools to use. These initiatives could not only improve the implementation of the CPS but also maximize its impact on the quality of consultations and the overall patient experience.

#### Explanation of the CPS and Its Usefulness

Adding a brief explanatory text to the CPS, distributing an informational sheet during consultations, or integrating it into clinic onboarding kits were recommended. Strengthening its connection with *Discutons Santé* was also suggested.

#### Clinician and Staff Involvement

The importance of involving clinicians and administrative staff to encourage patient use of the CPS was recognized by both patients and the manager. The manager noted that support from respected physicians could significantly impact its adoption, while patient testimonials about how the CPS improved their consultations could also persuade others to use it.

#### Promotional Tools

Patients recommended posters, social media, and phone wait-time messages to increase the visibility of the CPS and the *Discutons Santé* website. The manager suggested PowerPoint presentations, and volunteers in waiting rooms could introduce patients to the CPS and *Discutons Santé* without burdening staff.

## Discussion

### Evaluation of the Achievement of Objectives

Interviews and focus groups reveal that most patients found the CPS helpful in structuring their thoughts and preparing for walk-in consultations. These findings align with previous studies on preparation tools, such as question prompt lists [18,20,21]. However, even when patients perceived a personal benefit, many waited for clinicians to acknowledge or legitimize its use during the encounter.

This pattern reflects prior findings showing that patient preparation often depends on clinician encouragement [20,21]. It also echoes participatory medicine theories emphasizing that patients’ participation is shaped by implicit role expectations and by physicians’ legitimization of participatory practices, with patients often expecting clinicians to initiate or validate the use of such tools [40]. Although the CPS was designed primarily for patient use, these findings suggest that this purpose needs to be clearly communicated when introducing the tool.

Physicians preferred direct verbal interaction and were often unaware of the CPS. In high-flow walk-in clinics, clinicians face competing priorities and limited time [31]. These findings support an approach that does not require clinicians to formally review or manage the sheet; brief acknowledgment may nevertheless help legitimize patients’ preparation [20,21].

Earlier or electronic access may facilitate patient preparation, but digitalization alone would not address the relational and professional norms identified here. Patients may still hesitate to introduce the CPS when its optional purpose is unclear or because of deference to medical authority, while clinicians’ nonuse may reflect established routines. Thus, nonuse should not be understood solely as an implementation failure, but also as a practice embedded in the social organization of care.

Finally, although the CPS aims to enhance patient participation, interviews offer little exploration of this. The theories of Cegala et al [11,12,19] emphasize addressing the psychosocial dimensions of illness for appropriate treatment, but not all interviewed patients recognized the relevance of such questions. For example, several did not understand the usefulness of discussing their concerns, even though it seemed logical to them to be worried when seeking care. This may stem from insufficient explanation of the CPS’s purpose. Thus, despite the literature on its benefits, patients may not feel the need to share their concerns during consultations, assuming these are implicit. This may also reflect the idea that they do not see themselves as active partners with valid experiential knowledge.

### Problems With the Implementation of CPS

One of the main challenges identified in our study is the lack of information and support surrounding the implementation of the CPS, despite efforts being made to explain it by the research team. The implementation problems appear to reflect a mismatch between the research team’s assumptions and the clinic’s actual readiness. Previous experience with the *Discutons Santé* website may have led the team to overestimate familiarity with the CPS, while no standardized workflow was established for explaining, circulating, or acknowledging the tool. Implementation also coincided with the clinic’s accreditation period, which may have reduced attention to a new initiative.

Patients retained the choice to present the CPS, but the clinic had no clear process for explaining this option or responding when they did so. A predictable, brief acknowledgment when patients chose to share it could support its use without creating a mandatory review task. Participants also recommended providing the CPS in advance, consistent with prior studies [21]. Clinics could, therefore, standardize how the CPS is offered and explained while adapting the process to local realities.

The implementation challenges observed in our study reflect barriers identified in the literature. Lau et al [41] highlighted that factors such as institutional policies, organizational culture, and available resources play a crucial role in the adoption of innovations in primary care. These elements are particularly relevant in high-flow primary care clinical settings where pressure on resources and time can hinder the implementation of new tools aimed at improving patient preparation and engagement.

The findings can be interpreted through the complementary lenses of PACE and RE-AIM, which informed the design of the study. From a PACE perspective, the CPS appears to facilitate the “Prepare” component most directly and, to some extent, supports patients in asking, checking, and expressing themselves more clearly during the encounter. However, the findings also suggest that preparation alone does not guarantee active use of the tool during the consultation. When physicians did not acknowledge the CPS, many patients did not feel legitimized to mobilize it, which limited its contribution to a more reciprocal form of participation.

From a RE-AIM perspective, the study highlights that the main challenge was not only the perceived usefulness of the tool, but also its implementation into routine practice. Reach was substantial in that many patients received the CPS, but the findings suggest that receipt did not necessarily translate into meaningful uptake, especially when the purpose of the tool was not clearly explained or when time constraints limited its completion. Effectiveness, as perceived by participants, was mainly related to improved preparation, reduced stress, and better organization of concerns, but these benefits were diminished when the tool was not recognized during the consultation. Based on the data collected, adoption remained unclear due to lack of specific questions related to this part of the framework in the interview guide. Implementation was hindered by unclear circulation procedures, inconsistent explanation, and weak integration into the clinic workflow. Finally, participants’ suggestions regarding earlier access, clearer communication, and digital formats point to conditions that may support future maintenance, but only if clinician acknowledgment and organizational support are also strengthened. Taken together, these 2 frameworks help to show that the CPS may support patient preparation at the interactional level, while its sustained value depends on stronger uptake at the organizational and clinical levels.

### Research Limitations

This study has limitations. First, the 3-to-5-month gap between CPS use and interviews may have affected participants’ recall accuracy. Thus, future research should implement strategies to better consider the on-site use of participants and prioritize real-time data collection. Second, only 2 physicians were interviewed, making it difficult to assume that their perceptions are shared by most physicians in the walk-in clinic. Limited physician access during implementation also likely influenced physician engagement.

The AI-assisted comparison was used only as a supplementary data coverage check and not for interpretive sense-making.

Lastly, as the researchers conducted the study in a university clinic with a unique structure, results may not apply to other settings. Family medicine residents, who see many patients, were not interviewed. Additionally, the interviewed physicians supervised residents remotely, requiring careful interpretation of their insights.

### Actionable Lessons for Other Walk-in Clinics

Our findings suggest practical lessons for walk-in clinics and other high-flow primary care settings seeking to implement patient-preparation tools such as the CPS: (1) at the organizational level, clinics should establish a clear process for offering the CPS as an optional patient-preparation tool and making it available before the visit. It need not be automatically transmitted to clinicians or formally reviewed by them. (2) At the relational level, the CPS should help patients identify and prioritize what they want to discuss. When patients choose to use it, a brief acknowledgment (eg, “Thank you for completing the CPS”) may legitimize their preparation without adding a formal task for clinicians.

### Conclusions

This study suggests that the CPS may be most feasible when used primarily to support patient preparation in walk-in clinics. It can help patients identify and prioritize what they want to discuss without requiring clinicians to formally review or manage the sheet. When patients choose to share their priorities, brief clinician acknowledgment may help bring them into the conversation.

Clear explanations, earlier access, and feasible clinic processes may support implementation. The CPS was codeveloped with patients and physicians, while administrative staff contributed to implementation evaluation. Future refinements could be coproduced through joint review by patients, physicians, and administrative staff, integrating both content and implementation perspectives. The explanatory summary and electronic access, now under evaluation, will be reported in future studies.

## Supplementary material

10.2196/83489 Multimedia Appendix 1 Consultation preparation sheet, English version.

10.2196/83489 Multimedia Appendix 2 Interview guides.

10.2196/83489 Checklist 1 COREQ checklist.

## Abbreviations

COREQ: Consolidated Criteria for Reporting Qualitative Research
CPS: consultation preparation sheet
PACE: prepare, ask, check, and express
RE-AIM: Reach, Effectiveness, Adoption, Implementation, and Maintenance

## References

[1] (2005). Le rôle du médecin de famille en santé publique et capacité d’intervention en cas d’urgence document de réflexion [Report in French]. https://www.cfpc.ca/CFPC/media/Ressources/Politiques-en-mati%C3%A8re-de-sant%C3%A9/Role_Fam_Doc_Dec05_fr.pdf.

[2] Richard C, Lussier MT (2016). La Communication Professionnelle En Santé [Book in French].

[3] Pomey MP, Flora L, Karazivan P (2015). Le « Montreal model »: enjeux du partenariat relationnel entre patients et professionnels de la santé [Article in French]. Santé Publique.

[4] Roter DL, Hall JA (2009). Communication and adherence: moving from prediction to understanding. Med Care.

[5] Schneider J, Kaplan SH, Greenfield S, Li W, Wilson IB (2004). Better physician-patient relationships are associated with higher reported adherence to antiretroviral therapy in patients with HIV infection. J Gen Intern Med.

[6] Jackson JL, Chamberlin J, Kroenke K (2001). Predictors of patient satisfaction. Soc Sci Med.

[7] Richard C, Glaser E, Lussier MT (2017). Communication and patient participation influencing patient recall of treatment discussions. Health Expect.

[8] Hillen MA, de Haes HCJM, Stalpers LJA (2014). How can communication by oncologists enhance patients’ trust? An experimental study. Ann Oncol.

[9] Lim L, Chow P, Wong CY (2011). Doctor-patient communication, knowledge, and question prompt lists in reducing preoperative anxiety: a randomized control study. Asian J Surg.

[10] Street RL (2013). How clinician-patient communication contributes to health improvement: modeling pathways from talk to outcome. Patient Educ Couns.

[11] Cegala DJ, Marinelli T, Post D (2000). The effects of patient communication skills training on compliance. Arch Fam Med.

[12] Cegala DJ (2011). An exploration of factors promoting patient participation in primary care medical interviews. Health Commun.

[13] Henselmans I, Heijmans M, Rademakers J, van Dulmen S (2015). Participation of chronic patients in medical consultations: patients’ perceived efficacy, barriers and interest in support. Health Expect.

[14] Richard C, Lussier MT (2009). La littératie en santé, une compétence en mal de traitement [Article in French]. Pédagogie Médicale.

[15] Lu Q, Link E, Baumann E, Schulz PJ (2024). Linking patient-centered communication with cancer information avoidance: the mediating roles of patient trust and literacy. Patient Educ Couns.

[16] Driesenaar JA, van Dulmen S, van Weert JCM, Noordman J (2020). Patients’ evaluation of a preparatory online communication tool for older patients with cancer preceding chemotherapy. Cancer Nurs.

[17] Hibbard J (2017). Patient activation and health literacy: what’s the difference? How do each contribute to health outcomes. Stud Health Technol Inform.

[18] Arthur J, Pawate V, Lu Z (2023). Helpfulness of question prompt sheet for patient-physician communication among patients with advanced cancer: a randomized clinical trial. JAMA Netw Open.

[19] Cegala DJ, Street RL, Clinch CR (2007). The impact of patient participation on physicians’ information provision during a primary care medical interview. Health Commun.

[20] Jaks R, Guggiari E, De Gani SM, Nicca D (2023). Patients’ perspectives on the use of a newly developed “Patients’ Guide for Doctor’s Visit”: DocVISITguide. Int J Environ Res Public Health.

[21] Ramlakhan JU, Dhanani S, Berta WB, Gagliardi AR (2023). Optimizing the design and implementation of question prompt lists to support person-centred care: a scoping review. Health Expect.

[22] Lussier MT, Richard C, Diallo FB (2017). Un outil web pour soutenir le partenariat patients-soignants – Discutons Santé [Article in French]. Pédagogie Médicale.

[23] (2022). Question builder. Healthdirect.

[24] Linn AJ, Vervloet M, van Dijk L, Smit EG, Van Weert JCM (2011). Effects of eHealth interventions on medication adherence: a systematic review of the literature. J Med Internet Res.

[25] Lussier MT, Richard C, Binta Diallo F (2021). I am ready to see you now, Doctor! A mixed-method study of the Let’s Discuss Health website implementation in primary care. Health Expect.

[26] Lussier MT, Richard C, Glaser E, Roberge D (2016). The impact of a primary care e-communication intervention on the participation of chronic disease patients who had not reached guideline suggested treatment goals. Patient Educ Couns.

[27] Portrait numérique des générations [Article in French]. Académie de la transformation numérique (2023).

[28] Salahub C, Austin PC, Bai L (2024). Health care utilization after a visit to a within-group family physician vs a walk-in clinic physician. Ann Fam Med.

[29] Jones M (2000). Walk-in primary medical care centres: lessons from Canada. BMJ.

[30] Lapointe-Shaw L, Kiran T, Salahub C (2023). Walk-in clinic patient characteristics and utilization patterns in Ontario, Canada: a cross-sectional study. CMAJ Open.

[31] Terpou BA, Lapointe-Shaw L, Wang R (2024). A shifting terrain: understanding the perspectives of walk-in physicians on their roles amid worsening primary care access in Ontario, Canada. PLoS One.

[32] Bessert B, Oltrogge-Abiry JH, Peters PS (2023). Synergism of an urgent care walk-in clinic with an emergency department. Dtsch Arztebl Int.

[33] Tong A, Sainsbury P, Craig J (2007). Consolidated Criteria for Reporting Qualitative Research (COREQ): a 32-item checklist for interviews and focus groups. Int J Qual Health Care.

[34] Glasgow RE, Vogt TM, Boles SM (1999). Evaluating the public health impact of health promotion interventions: the RE-AIM framework. Am J Public Health.

[35] Braun V, Clarke V (2022). Conceptual and design thinking for thematic analysis. Qual Psychol.

[36] Braun V, Clarke V, Cooper H, Camic PM, Long DL, Panter AT, Rindskopf D, Sher KJ APA Handbook of Research Methods in Psychology, Vol 2: Research Designs: Quantitative, Qualitative, Neuropsychological, and Biological.

[37] Wachinger J, Bärnighausen K, Schäfer LN, Scott K, McMahon SA (2025). Prompts, pearls, imperfections: comparing ChatGPT and a human researcher in qualitative data analysis. Qual Health Res.

[38] Zhang H, Wu C, Xie J, Lyu Y, Cai J, Carroll JM (2023). Redefining qualitative analysis in the AI era: utilizing chatgpt for efficient thematic analysis. arXiv.

[39] Sakaguchi K, Sakama R, Watari T (2025). Evaluating ChatGPT in qualitative thematic analysis with human researchers in the Japanese clinical context and its cultural interpretation challenges: comparative qualitative study. J Med Internet Res.

[40] Plagg B, Engl A, Piccoliori G (2022). Mutual role expectations by patients and general practitioners-a mixed methods study on complementarity. Healthcare (Basel).

[41] Lau R, Stevenson F, Ong BN (2015). Achieving change in primary care—causes of the evidence to practice gap: systematic reviews of reviews. Implementation Sci.

