# Supplementary material for: Patient Preparation in Walk-in Clinics Through the Implementation of a Consultation Preparation Sheet: Qualitative Study
Source: J Particip Med. 2026 Oct 8;18:e83489. doi: 10.2196/83489 (PMC13648944; doi:10.2196/83489)
Supplement: Supplementary file 1 [file jopm-v18-e83489-s001.PDF]

PREPARING MY MEDICAL APPOINTMENT

Gender : ○ Man ○ Woman

For what reason(s) or symptom(s) are you visiting the clinic ?

____

Are you worried ?
(Circle a number)

0 1 2 3 4 5 6 7 8 9 10
Not at all ... Very worried

If yes, specify : ____

What do you expect from you appointment today ?

○ I want to understand what these symptoms are
○ I want to be relieved
○ I need a leave from work
○ I need a prescription
○ I would like to pass some tests
○ Other : ______________________________

How much discomfort do you have ? (Circle a number, a face or a word)

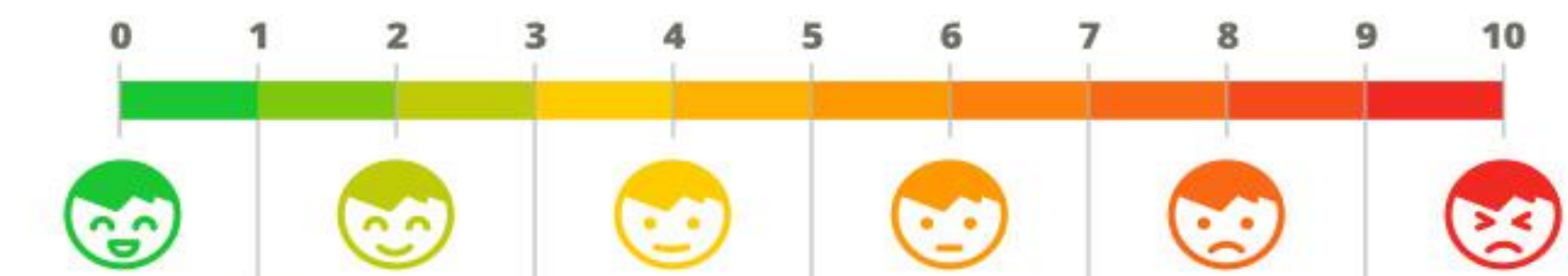

No discomfort | Light discomfort | Moderate discomfort | Intense discomfort | Horrible discomfort | Unbearable discomfort

About your problem

Is the discomfort persistant ? ○ Yes ○ No

Have you tried something to relieve your discomfort ? ○ Yes ○ No

If yes, specify : Does it work ?

1. ____ ○ Yes ○ No ○ A little bit
2. ____ ○ Yes ○ No ○ A little bit
3. ____ ○ Yes ○ No ○ A little bit

Have you been hospitalized for this problem ? ○ Yes ○ No

Have you ever consulted someone for this problem ? ○ Yes ○ No

If yes, specify :

○ Here (Cité de la Santé Family Medicine Group)
○ Another doctor
○ Emergency room of Cité de la Santé
○ Another emergency room
○ Other, specify : ____

V.06.02.2019

About your symptoms

Describe all of your symptoms and when they first appeared.

**Symptoms**	**When they first appeared**			
Example : pain at the right ear	*____ hour(s)*	*__3_ day(s)*	*____ week(s)*	*____ months*
______________________________	____ hour(s)	____ day(s)	____ week(s)	____ months
______________________________	____ hour(s)	____ day(s)	____ week(s)	____ months
______________________________	____ hour(s)	____ day(s)	____ week(s)	____ months
______________________________	____ hour(s)	____ day(s)	____ week(s)	____ months

Indicate where your symptoms are located.
Put an X or circle the region on the picture.

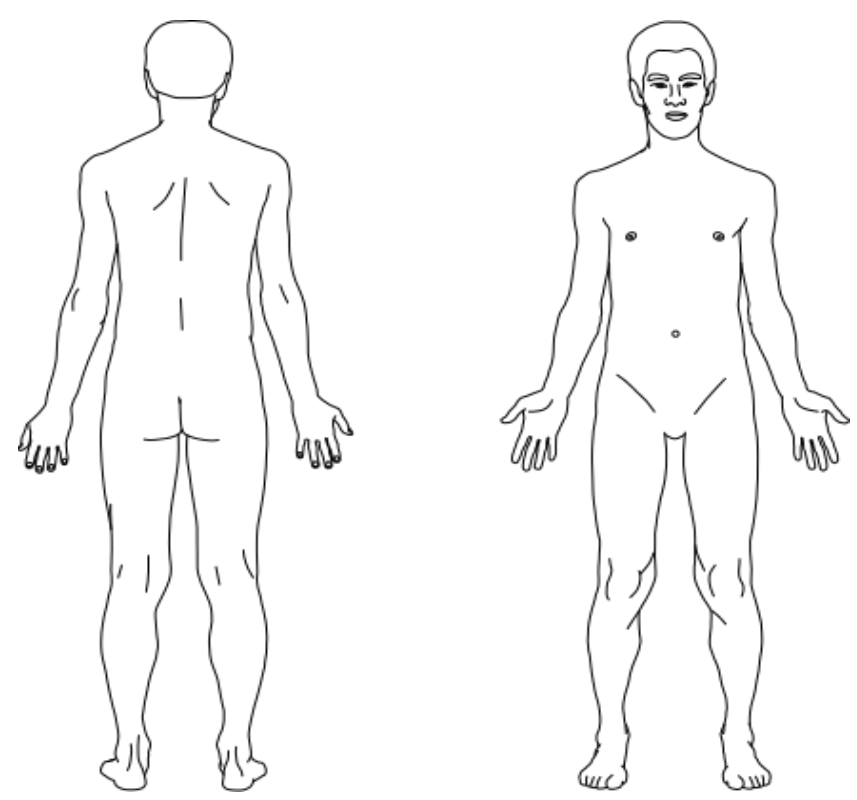

With this problem, I cannot or it is difficult for me to :

○ Sleep ○ Eat ○ Drink ○ Breath ○ Walk ○ Work ○ Practising sports

○ Other, specify : ____

Do you have an idea of what may be causing your problem ? ○ Yes ○ No

____

____

What other important question(s) would you like to ask to the doctor ?

____

____

When you will be called to a medical examination room :

1. Take off your jacket or coat.
2. Medication list : Have your medication list on hand. We may ask for it.
3. This document : Keep it with you. You may give it to the auxiliary nurse or the doctor during the consultation.
