# Supplementary material for: Patient Preparation in Walk-in Clinics Through the Implementation of a Consultation Preparation Sheet: Qualitative Study
Source: J Particip Med. 2026 Oct 8;18:e83489. doi: 10.2196/83489 (PMC13648944; doi:10.2196/83489)
Supplement: Supplementary file 2 [file jopm-v18-e83489-s002.pdf]

DISCUTONS SANTÉ PROJECT

EMERGENCY ROOM, WALK-IN CLINIC, SPECIALIZED CLINICS

V.25.11.2019

FOCUS GROUPS OR SEMI-STRUCTURED INTERVIEW GUIDE

PATIENTS

Instructions for the facilitator:

- Make sure to start the audio recorder to record the discussions.
- Below is a list of themes to discuss with participants.
- For each theme, a list of suggested questions is provided to facilitate the discussion if needed.
- Note: some questions are relevant only for certain clinics.
- Duration of the focus group: ~ 1 hour
- Abbreviation: DS = Discutons Santé; CPS = Consultation Preparation Sheet

DURATION	THEMES AND SUGGESTED QUESTIONS
15 min	• Welcome participants, sign consent form, distribute lunch boxes<br>• Round-table introductions<br>• Presentation of the different themes to be discussed
15 min	**USE OF DS (WEBSITE, CPS)**<br><br>Q1 How did you hear about DS (telephone message, appointment confirmation email, bookmarks, posters, consultation preparation sheet, etc.)?<br><br>*For specialized clinics only:*<br>Q2 What was your reaction when you received the invitation to visit the DS website to prepare for your medical visit (e.g., interest, curiosity, enthusiasm, reluctance, mistrust, etc.)? Please explain.<br>Q3 Why did you decide to visit the DS website?<br>Q4 How did you find the process of preparing for your medical visit using the DS website?<br>a. Did you find it time-consuming to prepare for your medical visit?<br>b. Did you encounter any difficulties using DS to prepare for your medical visit? If yes, please explain.<br>c. Did the Summary form suit your needs? Compared to your usual way of preparing, what are its advantages and disadvantages<br>Q5 Did you consult…<br>a. the PACE skills section? What are your impressions?<br>b. the health notebook? What are your impressions?<br>c. the disease information sections? Which one(s)? What are your impressions?<br><br>*(continued on reverse side)*<br><br>**USE OF DS (WEBSITE, CPS) (continued)**<br><br>Q6 In your opinion, is anything missing from the DS website (including PACE modules, health notebook, disease information sections)? Please explain.<br><br>*For emergency and walk-in clinics only:*<br>Q7 What was your reaction when you were asked to complete the CPS?<br>Q8 Did you find it difficult/demanding/time-consuming to complete the form?<br>Q9 Were there any questions that you…<br>a. … appreciated (e.g., expectations, worries, disruption of daily activities, etc.)?<br>b. … found unnecessary? Please specify.<br>Q10 Did you have enough time to complete it before seeing the doctor?

	Q11 After your medical visit, did you visit the DS website? If yes, what are your impressions?
15 min	**IMPACT OF DS/CPS ON THE MEDICAL CONSULTATION**<br>Q12 Did you share the content of the CPS with the doctor?<br>Q13 At what point did you share your CPS with the doctor?<br>a. Did the doctor ask whether you had it?<br>b. If not, were you surprised or disappointed?<br>Q14 Were you comfortable sharing the content of the CPS with the doctor? Please explain.<br>Q15 How did the doctor react when you gave her the CPS?<br>a. Did the doctor take the CPS into account during the consultation?<br>b. Did the doctor's reaction meet your expectations?<br>Q16 Were you able to…<br>a. … describe your symptoms/health problem(s) or present the topics you wanted to discuss?<br>b. … express your concerns/worries about your health?<br>c. … ask questions during the consultation?<br>Q17 Did you think about checking the information provided by the doctor?<br>Q18 In your opinion, what are the advantages of preparing for your medical visit using the DS website or the CPS?<br>a. Not forgetting anything you wanted to discuss?<br>b. Managing/reducing stress related to the medical visit?<br>c. Effects on the appointment agenda?<br>d. Effects on the role you usually play during the consultation?<br>e. Effects on your satisfaction with the appointment?
10 min	**SUGGESTIONS AND COMMENTS**<br>Q19 Now that you have used the CPS, do you have any suggestions to improve appointment preparation (e.g., questions asked, missing information, etc.)?<br>Q20 Do you plan to use DS or the CPS to prepare for your future medical visits?<br>a. If not, please explain.<br>Q21 For what type(s) of appointments would the CPS be most useful to you (e.g., regular follow-up appointments, walk-in clinic visits, emergency room visits, etc.)?<br>Q22 Under what circumstances would the CPS be most useful to you (e.g., unfamiliar healthcare professional, when it has been a long time since seeing your healthcare professional, new health problem, known health problem that is worsening, etc.)?<br>Q23 Have you talked about DS with people around you (parents, children, siblings, friends, etc.)? Do you plan to do so?<br>a. Would you recommend that they use it to prepare for their appointments?<br>Q24 Do you have any suggestions for promoting the CPS among patients at your clinic?

DISCUTONS SANTÉ PROJECT

EMERGENCY ROOM, WALK-IN CLINIC, SPECIALIZED CLINICS

V.25.11.2019

FOCUS GROUPS OR SEMI-STRUCTURED INTERVIEW GUIDE

PATIENTS

Instructions for the facilitator:

- Make sure to start the audio recorder to record the discussions.
- Below is a list of themes to discuss with participants.
- For each theme, a list of suggested questions is provided to facilitate the discussion if needed.
- Note: some questions are relevant only for certain clinics.
- Duration of the focus group: ~ 1 hour
- Abbreviation: DS = Discutons Santé; CPS = Consultation Preparation Sheet

<table>
<tr><th>DURATION</th><th>THEMES AND SUGGESTED QUESTIONS</th></tr>
<tr><td>15 min</td><td>• Welcome participants, sign consent form, distribute lunch boxes
• Round-table introductions
• Presentation of the different themes to be discussed</td></tr>
<tr><td>15 min</td><td>USE OF DS (WEBSITE, CPS)

Q1 How did you hear about DS (telephone message, appointment confirmation email, bookmarks, posters, consultation preparation sheet, etc.)?

For specialized clinics only:
Q2 What was your reaction when you received the invitation to visit the DS website to prepare for your medical visit (e.g., interest, curiosity, enthusiasm, reluctance, mistrust, etc.)? Please explain.
Q3 Why did you decide to visit the DS website?
Q4 How did you find the process of preparing for your medical visit using the DS website?
a. Did you find it time-consuming to prepare for your medical visit?
b. Did you encounter any difficulties using DS to prepare for your medical visit? If yes, please explain.
c. Did the Summary form suit your needs? Compared to your usual way of preparing, what are its advantages and disadvantages
Q5 Did you consult…
a. the PACE skills section? What are your impressions?
b. the health notebook? What are your impressions?
c. the disease information sections? Which one(s)? What are your impressions?

(continued on reverse side)

USE OF DS (WEBSITE, CPS) (continued)

Q6 In your opinion, is anything missing from the DS website (including PACE modules, health notebook, disease information sections)? Please explain.

For emergency and walk-in clinics only:
Q7 What was your reaction when you were asked to complete the CPS?
Q8 Did you find it difficult/demanding/time-consuming to complete the form?
Q9 Were there any questions that you…
a. … appreciated (e.g., expectations, worries, disruption of daily activities, etc.)?
b. … found unnecessary? Please specify.
Q10 Did you have enough time to complete it before seeing the doctor?</td></tr>
</table>

<table>
<tr><td></td><td>Q11 After your medical visit, did you visit the DS website? If yes, what are your impressions?</td></tr>
<tr><td>15 min</td><td>IMPACT OF DS/CPS ON THE MEDICAL CONSULTATION
Q12 Did you share the content of the CPS with the doctor?
Q13 At what point did you share your CPS with the doctor?
a. Did the doctor ask whether you had it?
b. If not, were you surprised or disappointed?
Q14 Were you comfortable sharing the content of the CPS with the doctor? Please explain.
Q15 How did the doctor react when you gave her the CPS?
a. Did the doctor take the CPS into account during the consultation?
b. Did the doctor’s reaction meet your expectations?
Q16 Were you able to…
a. … describe your symptoms/health problem(s) or present the topics you wanted to discuss?
b. … express your concerns/worries about your health?
c. … ask questions during the consultation?
Q17 Did you think about checking the information provided by the doctor?
Q18 In your opinion, what are the advantages of preparing for your medical visit using the DS website or the CPS?
a. Not forgetting anything you wanted to discuss?
b. Managing/reducing stress related to the medical visit?
c. Effects on the appointment agenda?
d. Effects on the role you usually play during the consultation?
e. Effects on your satisfaction with the appointment?</td></tr>
<tr><td>10 min</td><td>SUGGESTIONS AND COMMENTS
Q19 Now that you have used the CPS, do you have any suggestions to improve appointment preparation (e.g., questions asked, missing information, etc.)?
Q20 Do you plan to use DS or the CPS to prepare for your future medical visits?
a. If not, please explain.
Q21 For what type(s) of appointments would the CPS be most useful to you (e.g., regular follow-up appointments, walk-in clinic visits, emergency room visits, etc.)?
Q22 Under what circumstances would the CPS be most useful to you (e.g., unfamiliar healthcare professional, when it has been a long time since seeing your healthcare professional, new health problem, known health problem that is worsening, etc.)?
Q23 Have you talked about DS with people around you (parents, children, siblings, friends, etc.)? Do you plan to do so?
a. Would you recommend that they use it to prepare for their appointments?
Q24 Do you have any suggestions for promoting the CPS among patients at your clinic?</td></tr>
</table>

DISCUTONS SANTÉ PROJECT

EMERGENCY ROOM, WALK-IN CLINIC, SPECIALIZED CLINICS

June 19, 2019 – Version 3

SEMI-STRUCTURED INTERVIEW GUIDE

MANAGER

Instructions for the facilitator:

- Make sure to start the audio recorder to record the discussions.
- Below is a list of themes to discuss. For each theme, a list of suggested questions is provided to facilitate the discussion if needed.
- Note: some questions are relevant only for certain clinics.
- Duration of the semi-structured interview: 1 hour
- Abbreviation: DS = Discutons Santé; CPS = Consultation Preparation Sheet

DURATION	THEMES AND SUGGESTED QUESTIONS
2 min	• Welcome participant, sign consent form and present the different themes to be discussed
25 min	**INTEGRATION OF THE CPS INTO THE ORGANIZATION OF WORK**<br>Q1 How did the integration of the CPS go?<br>a. Did you encounter any difficulties? If yes, please explain.<br>b. Did you identify any factors that facilitated the integration of the CPS? If yes, which ones?<br>Q2 Did you receive any feedback from your clinicians regarding the integration of the CPS? If yes, what feedback?<br>Q3 Did you need to support your clinicians in integrating the CPS? If yes, describe the type of support requested. Please describe what was put in place.<br>***DS/CPS promotional tools***<br>Q4 What do you think of the DS/CPS promotional tools?<br>Q5 How were these tools useful to you in integrating DS/CPS?<br>Q6 How was the management of promotional tools handled (place of distribution/access to promotional tools, inventory management, scripts, etc.)?<br>Q7 Were responsibilities/tasks assigned to certain employees to manage the promotional tools? If yes…<br>a. … did you encounter any difficulties? If yes, please explain.<br>b. … did you identify any facilitating factors? If yes, which ones?
15 min	**IMPACT OF THE CPS ON THE ORGANIZATION OF THE CLINIC**<br>Q8 Did the integration of the CPS affect staff workload? If yes, in what way?<br>Q9 What adaptations in the organization of work were required (welcoming users, distributing and collecting the CPS material)? How was this put in place?<br>a. Did you encounter any difficulties? If yes, please explain.<br>b. Did you identify any facilitating factors? If yes, which ones?<br>Q10 How were these adaptations experienced by your staff?
5 min	**SUGGESTIONS AND COMMENTS**<br>Q11 Do you intend to continue using the CPS?<br>a. If yes, what type of support do you need (long-term management of promotional tools, adaptation of current promotional tools, training, etc.)?<br>b. If not, for what reason(s)?

DISCUTONS SANTÉ PROJECT

EMERGENCY ROOM, WALK-IN CLINIC, SPECIALIZED CLINICS

June 19, 2019 – Version 3

FOCUS GROUP INTERVIEW GUIDE

ADMINISTRATIVE STAFF

Instructions for the facilitator:

- Make sure to start the audio recorder to record the discussions.
- Below is a list of themes to discuss with participants.
- For each theme, a list of suggested questions is provided to facilitate the discussion if needed.
- Note: some questions are relevant only for certain clinics.
- Duration of the focus group: 1 hour
- Abbreviation: DS = Discutons Santé ; CPS = Consultation Preparation Sheet

DURATION	THEMES AND SUGGESTED QUESTIONS
15 min	• Welcome participants, sign consent form, distribute lunch boxes<br>• Round-table introductions<br>• Presentation of the different themes to be discussed
20 min	**INTEGRATION OF DS TOOLS INTO ADMINISTRATIVE ROUTINES**<br><br>Q1 To what extent did using the CPS affect your workload?<br><br>*For specialized clinics only:*<br>Q2 How did you perceive the task of inviting users to use DS to prepare for their consultation…<br>a. … by telephone?<br>b. … by email?<br>c. … when they arrived at the clinic reception desk?<br><br>*For emergency room and walk-in clinics only:*<br><br>Q3 How did you perceive the task of inviting users to complete the CPS when they arrived at the clinic?<br><br>*For all clinics:*<br>Q4 To what extent were you comfortable answering users' questions about DS/ the CPS? Did you feel that it was your role to provide this information?<br>Q5 Did you encounter any difficulties integrating DS/CPS into your work routine? If yes, please explain.<br>Q6 Did you identify any factors that facilitated the integration of DS/ the CPS into your work routine? If yes, which ones (e.g., having promotional tools, sample scripts, receiving training on DS/CPS at the beginning of the project, having the manager's support, etc.)?
10 min	**USERS' RESPONSES FOLLOWING THE INVITATION TO VISIT DS/CPS**<br><br>Q7 In your opinion, to what extent were users open to visiting the DS website or completing the CPS to prepare for their medical consultation?<br>Q8 In your opinion, what were the facilitating factors (e.g., the website was free, simple to use, available tools [communication tips, appointment preparation process, health notebook], etc.)?<br>Q9 In your opinion, what were the limiting factors (e.g., no access to the Internet/a computer/a printer, not comfortable navigating the Internet, concerns about sharing personal data, etc.)?
10 min	**SUGGESTIONS AND COMMENTS**<br><br>Q10 Do you have any suggestions for continuing to promote DS/ the CPS among users of your clinic?<br>Q11 To what extent are you comfortable continuing to promote DS/ the CPS to…<br>a. … users consulting your service?<br>b. … your colleagues?<br>c. … clinicians in your service?

	Q12 In your opinion, what procedure(s) should be implemented, or what document(s) should be available, to inform and train “new” staff or replacement staff about DS / the CPS?
